# Trends in the Prevalence of Excess Dietary Sodium Intake — United States, 2003–2010

**Published:** 2013-12-20

**Authors:** Alicia Carriquiry, Alanna J. Moshfegh, Lois C. Steinfeldt, Mary E. Cogswell, Fleetwood Loustalot, Zefeng Zhang, Quanhe Yang, Niu Tian

**Affiliations:** Iowa State Univ; Food Surveys Research Group, Beltsville Human Nutrition Research Center, Agricultural Research Svc, US Dept of Agriculture; Div for Heart Disease and Stroke Prevention, National Center for Chronic Disease Prevention and Health Promotion; EIS Officer, CDC

Excess sodium intake can lead to hypertension, the primary risk factor for cardiovascular disease, which is the leading cause of U.S. deaths (1). Monitoring the prevalence of excess sodium intake is essential to provide the evidence for public health interventions and to track reductions in sodium intake, yet few reports exist. Reducing population sodium intake is a national priority, and monitoring the amount of sodium consumed adjusted for energy intake (sodium density or sodium in milligrams divided by calories) has been recommended because a higher sodium intake is generally accompanied by a higher calorie intake from food (2). To describe the most recent estimates and trends in excess sodium intake, CDC analyzed 2003–2010 data from the National Health and Nutrition Examination Survey (NHANES) of 34,916 participants aged ≥1 year. During 2007–2010, the prevalence of excess sodium intake, defined as intake above the Institute of Medicine tolerable upper intake levels (1,500 mg/day at ages 1–3 years; 1,900 mg at 4–8 years; 2,200 mg at 9–13 years; and 2,300 mg at ≥14 years) (3), ranged by age group from 79.1% to 95.4%. Small declines in the prevalence of excess sodium intake occurred during 2003–2010 in children aged 1–13 years, but not in adolescents or adults. Mean sodium intake declined slightly among persons aged ≥1 year, whereas sodium density did not. Despite slight declines in some groups, the majority of the U.S. population aged 1 year consumes excess sodium.

NHANES is a nationally representative, multistage survey of the noninstitutionalized U.S. civilian population. Certain populations are oversampled to allow for reliable estimates within subgroups.* During NHANES 2003–2010, a total of 49,731 participants aged ≥1 year (including those currently breastfed) were screened. Participants who completed an initial in-person dietary recall in a mobile examination center were asked to complete a second 24-hour dietary recall by telephone 3–10 days later. After those with missing or incomplete dietary recall data were excluded, the final analytic sample was 34,916, for a response rate of 70.3% among those screened. The 24-hour dietary recall was collected by trained interviewers using the U.S. Department of Agriculture (USDA) automated multiple-pass method† by proxy for those aged 1–5 years, by participants with proxy assistance for those aged 6–11 years, and directly by participants aged ≥12 years. The nutrient values of sodium were assigned to foods and beverages using the USDA Food and Nutrient Database for Dietary Studies corresponding with each NHANES 2-year cycle.§ Sodium intake for each respondent on each recall day was estimated by summing the sodium consumed from each food and beverage during the previous 24 hours (excluding supplements, antacids, and salt added at the table). To evaluate trends, from 2003–2010, estimates of sodium in foods did not include salt adjustments for participants whose household used salt in cooking occasionally or less often.¶ For children consuming human milk, the sodium content was estimated and added to sodium from other foods and beverages.**

Up to two 24-hour dietary recalls were used. Data were analyzed with statistical software that fits a measurement error model.†† All estimates were based on usual sodium intake, adjusting for within person, day-to-day variability. After adjusting for the day of the week of the recall, age (years), sex, and race/ethnicity, estimates were calculated for mean usual sodium intake, sodium density, and prevalence of excess sodium intake. Jackknife replicate weights based on survey weights were used to estimate standard errors and account for the complex survey design. The differences in the prevalence of excess sodium intake were examined by z test. Using linear regression models with the usual mean intake for each 2-year phase weighted by the inverse of the variance, trends in sodium intake and sodium intake density were examined using a z test. A p-value of <0.05 was considered statistically significant. No adjustment was made for multiple testing.

What is already known on this topic?

Excess sodium intake can lead to hypertension and consequent cardiovascular disease. Sodium consumption in the United States is well above national recommendations. Reports of national data on sodium consumption trends are limited.

What is added by this report?

As of 2010, >90% of U.S. adolescents and adults consume sodium in excess of recommendations, and little has changed since 2003. U.S. children have seen a slight decline in excess sodium consumption during the same period, but 80%–90% of children continue to consume excess sodium. From 2003 to 2010, a slight decrease occurred in average sodium intake, but not sodium intake per calorie.

What are the implications for public health practice?

Small reductions in sodium intake might be related to declines in average energy consumption, rather than changes in the amount of sodium per calorie in foods consumed. Given that average energy and sodium intakes have changed little over time, coupling efforts to reduce obesity with efforts to reduce the sodium content per calorie in foods might accelerate reductions in sodium consumed.

During 2007–2010, the prevalence of excess usual sodium intake ranged from 79.1% for U.S. children aged 1–3 years to 95.4% for U.S. adults aged 19–50 years (Table 1). A statistically significant 2.7–4.9 percentage point decline in excess usual sodium intake occurred from 2003–2006 to 2007–2010 among children aged 1–3, 4–8, and 9–13 years, but not among adolescents or adults. Among children aged 4–8 years, statistically significant declines occurred across all sex and race/ethnicity subgroups.

Mean usual sodium intake among the U.S. population aged ≥1 year decreased slightly from 2003–2004 to 2009–2010 (3,518 mg versus 3,424 mg; p-value for trend = 0.037). The U.S. population aged ≥1 year consumed, on average, approximately 1,700 mg sodium per 1,000 kcal during 2009–2010, with no significant trend over time compared with previous investigation years (Table 2). Across age groups, mean usual sodium density did not change significantly over time, with the exception of youths aged 14–18 years, for whom sodium density increased slightly. Within age groups, mean usual sodium density slightly increased among males aged 4–8 years and females aged 14–18 years and slightly declined among non-Hispanic whites aged ≥51 years.

## Editorial Note

The findings in this report indicate that during 2007–2010, approximately eight out of 10 U.S. children aged 1–3 years and nine out of 10 U.S. residents aged ≥4 years were at potential risk for high blood pressure attributable to excess sodium intake. Although a slight decrease in the prevalence of excess usual sodium intake occurred after 2003–2006 among children aged 1–13 years, excess intake did not decrease among adolescents and adults. During 2003–2010, a slight decrease occurred in average population sodium intake, but not sodium intake per calorie. Although some variation in trends occurred among population subgroups in usual mean sodium intake and sodium density, the lack of a change in sodium consumed per calorie (approximately 1,700 mg/1,000 kcal) suggests that the small reduction in usual sodium intake might be related to declines in calorie consumption, rather than to changes in sodium density of foods.

Previous reports (4,5) included data on trends in U.S. sodium intake from the 1970s to 2003. The findings in this report update these trends, and include new data on usual excess sodium intake and sodium density. The slight declines in excess usual sodium intake among children aged 1–13 years might be partially explained by declines in energy intake among children over the same period.§§ Given an average sodium consumption of 1,700 mg/1,000 kcal/day, reducing 100 calories per day could result in a mean reduction of 170 mg of sodium per day, slightly shifting the distribution of sodium intake and lowering the percentage of those with excess intake. Among adults, the pattern of trends in sodium intake also might be explained by changes in energy intake over time. Although average energy intake declined slightly during 1999–2010 among adults aged 20–39 years, it did not change among older adults (6).

The findings in this report are subject to at least four limitations. First, NHANES data exclude military personnel and institutionalized populations such as persons who reside in long-term care or correctional facilities. Second, the response rate was 70.3%; lower response rates can result in response bias. Third, the 24-hour dietary recall underestimates mean caloric intake by an estimated 11% and sodium intake by 9%, and sodium intake excluded use of salt at the table, which accounts for nearly 5% of U.S. sodium intake (7). Finally, no adjustments for multiple comparisons were performed to determine whether differences between any pair of estimates were statistically significant.

Despite slight declines in sodium intake among some population groups, most U.S. residents aged ≥1 year consume excess sodium. Given consumption of approximately 1,700 mg of sodium per 1,000 kilocalories/day, a mean energy reduction of approximately 600 kcal/day would be required to reduce mean sodium intake by approximately 1,000 mg, to approximately 2,300 mg/day. A sodium density target of 1,000 mg/1,000 kcal was recently proposed to lower sodium intake to <2,300 mg per day (2). Given that average energy and sodium intakes have changed little over time, coupling efforts to reduce obesity with efforts to reduce the sodium content per calorie in foods might accelerate progress. Considering that 8.1% of sodium intake among U.S. children comes from school meals (8), new school food guidelines might promote progress toward achieving goals for reducing sodium consumption among children who obtain meals at school.¶¶ Other ongoing public health efforts include working with industry to gradually reduce sodium in commercially processed packaged and restaurant foods.*** Even a 400 mg reduction in mean U.S. sodium intake might save billions of health-care dollars (9).

## Figures and Tables

**TABLE 1:** Proportion of usual sodium intake exceeding the Institute of Medicine tolerable upper intake level,* by age group, sex, and race/ethnicity† — National Health and Nutrition Examination Survey (NHANES), United States, 2003–2010

Characteristic	Upper limit (mg/day)	2003–2006			2007–2010			Percentage point change	p-value
	
		No.§	Proportion over upper intake level (%)	Standard error	No.	Proportion over upper intake level (%)	Standard error		
**Age 1–3 yrs**	1,500	1,560	(84.0)	1.4	1,558	(79.1)	1.9	(−4.9)	0.019¶
Male		784	(84.1)	2.0	809	(79.4)	2.7	(−4.7)	0.081
Female		776	(84.3)	2.2	749	(79.7)	2.2	(−4.6)	0.071
White, non-Hispanic		470	(84.0)	2.9	525	(80.3)	3.7	(−3.7)	0.215
Black, non-Hispanic		407	(87.6)	3.3	297	(86.3)	3.0	(−1.3)	0.385
Mexican-American		519	(75.7)	3.2	437	(71.2)	4.9	(−4.5)	0.222
**Age 4–8 yrs**	1,900	1,682	(97.3)	0.4	1,890	(92.6)	0.8	(−4.6)	<0.001¶
Male		815	(97.7)	0.5	995	(94.3)	1.0	(−3.4)	0.008¶
Female		867	(96.9)	0.8	895	(90.5)	1.4	(−6.3)	<0.001¶
White, non-Hispanic		479	(96.3)	0.8	621	(90.3)	1.5	(−5.9)	<0.001¶
Black, non-Hispanic		519	(98.9)	0.7	402	(95.6)	1.3	(−3.3)	0.012¶
Mexican-American		517	(94.2)	1.4	529	(89.3)	2.6	(−4.9)	0.045¶
**Age 9–13 yrs**	2,200	2,040	(96.9)	0.7	1,717	(94.2)	0.9	(−2.7)	0.008¶
Male		999	—**	—**	850	(96.8)	0.7	—††	—††
Female		1,041	(91.4)	1.6	867	(90.1)	1.7	(−1.4)	0.279
White, non-Hispanic		516	(97.0)	0.8	544	—**	—**	—††	—††
Black, non-Hispanic		691	—**	—**	406	—**	—**	—††	—††
Mexican-American		669	(95.4)	1.3	456	(84.8)	3.1	(−10.5)	0.001¶
**Age 14–18 yrs**	2,300	2,673	(94.2)	1.0	1,552	(92.3)	1.5	(−1.9)	0.145
Male		1,353	(97.8)	0.7	818	—**	—**	—††	—††
Female		1,320	(84.2)	2.3	734	(80.2)	3.1	(−4.0)	0.938
White, non-Hispanic		731	(95.7)	1.0	517	(93.4)	1.7	(−2.3)	0.123
Black, non-Hispanic		938	(90.7)	1.8	369	—**	—**	—††	—††
Mexican-American		820	(94.3)	1.3	385	(90.0)	2.2	(−4.3)	0.047¶
**Age 19–50 yrs**	2,300	5,428	(95.9)	0.4	6,086	(95.4)	0.5	(−0.5)	0.200
Male		2,528	(99.2)	0.1	2,936	(99.1)	0.2	(−0.1)	0.242
Female		2,900	(86.6)	1.2	3,150	(84.8)	1.4	(−1.9)	0.152
White, non-Hispanic		2,384	(97.1)	0.4	2,598	(96.4)	0.6	(−0.7)	0.170
Black, non-Hispanic		1,310	(92.5)	1.4	1,190	(93.4)	0.8	(0.9)	0.709
Mexican-American		1,276	(93.5)	1.0	1,270	(90.8)	1.3	(−2.8)	0.050
**Age ≥51 yrs**	2,300	4,062	(88.9)	1.0	4,668	(90.1)	0.8	(1.2)	0.839
Male		2,028	(95.9)	0.6	2,341	(96.5)	0.5	(0.6)	0.782
Female		2,034	(77.1)	1.4	2,327	(77.9)	1.4	(0.9)	0.668
White, non-Hispanic		2,416	(91.4)	0.9	2,273	(92.8)	0.8	(1.4)	0.876
Black, non-Hispanic		762	(79.0)	2.4	975	(82.2)	2.0	(3.2)	0.842
Mexican-American		674	(67.7)	3.9	757	(76.3)	3.1	(8.6)	0.959

* The upper intake level is the age-specific, tolerable upper intake level, as defined by the Institute of Medicine (2005). The proportion of usual sodium intake over the upper intake level was estimated using PC-SIDE software (Department of Statistics, Iowa State University) with jackknife replicate weights and adjusted for the day of the week of the recall, age (years), sex, and race/ethnicity. Persons missing data on incomplete first-day recall were excluded from the analysis.

† Other racial/ethnic groups were not included. The sum of the sample size of non-Hispanic white, non-Hispanic black, and Mexican-American is not equal to the total sample size.

§ Sample sizes unweighted.

¶ p<0.05, when trends of proportion of usual sodium intake over the upper intake level were examined using the z test.

** Data statistically unreliable; relative standard error ≥0.3.

†† Not applicable.

**TABLE 2:** Mean usual sodium density* (mg/1,000 kcal), by age group, sex, and race/ethnicity† — National Health and Nutrition Examination Survey (NHANES), United States, 2003–2010

Characteristic	2003–2004			2005–2006			2007–2008			2009–2010			Changes per cycle¶	p-value for trend
			
	No.§	Mean	Standard error	No.	Mean	Standard error	No.	Mean	Standard error	No.	Mean	Standard error		
**Overall**	**8,579**	**1,661**	**10**	**8,866**	**1,693**	**14**	**8,473**	**1,697**	**12**	**8,998**	**1,689**	**10**	**9**	**0.248**
Male	4,192	1,653	9	4,315	1,666	14	4,266	1,695	15	4,483	1,690	14	14	0.054
Female	4,387	1,669	17	4,551	1,719	17	4,207	1,698	16	4,515	1,688	13	2	0.879
White, non-Hispanic	3,541	1,679	10	3,455	1,710	14	3,367	1,698	11	3,711	1,692	10	4	0.560
Black, non-Hispanic	2,284	1,617	26	2,343	1,637	14	1,939	1,664	21	1,700	1,632	17	5	0.652
Mexican-American	2,123	1,548	15	2,352	1,569	16	1,773	1,582	16	2,061	1,581	28	13	0.063
**Age 1–3 yrs**	740	1,431	21	820	1,458	34	765	1,429	23	793	1,427	15	−3	0.589
Male	363	1,404	32	421	1,472	46	399	1,392	34	410	1,419	25	0	0.993
Female	377	1,457	31	399	1,433	20	366	1,463	27	383	1,433	22	−3	0.727
White, non-Hispanic	226	1,435	25	244	1,472	36	246	1,399	36	279	1,434	34	−5	0.729
Black, non-Hispanic	218	1,500	30	189	1,464	34	163	1,497	29	134	1,479	75	−3	0.840
Mexican-American	228	1,364	49	291	1,343	31	207	1,368	46	230	1,360	47	3	0.695
**Age 4–8 yrs**	783	1,541	19	899	1,550	19	934	1,530	20	956	1,556	23	2	0.822
Male	382	1,491	20	433	1,531	21	500	1,544	31	495	1,573	41	27	0.028**
Female	401	1,594	29	466	1,567	28	434	1,518	24	461	1,541	21	−18	0.252
White, non-Hispanic	220	1,545	31	259	1,522	28	300	1,480	26	321	1,546	37	−7	0.747
Black, non-Hispanic	261	1,574	42	258	1,614	40	230	1,620	32	172	1,568	32	−3	0.840
Mexican-American	224	1,434	34	293	1,491	23	250	1,524	31	279	1,487	31	3	0.695
**Age 9–13 yrs**	995	1,601	23	1,045	1,633	16	832	1,637	32	885	1,636	19	9	0.292
Male	482	1,580	35	517	1,640	29	411	1,647	40	439	1,665	30	25	0.102
Female	513	1,622	34	528	1,627	39	421	1,625	45	446	1,613	27	−3	0.269
White, non-Hispanic	266	1,568	28	250	1,648	25	252	1,638	45	292	1,635	23	17	0.370
Black, non-Hispanic	350	1,750	58	341	1,685	39	224	1,722	48	182	1,599	30	−44	0.140
Mexican-American	301	1,520	45	368	1,613	27	206	1,514	64	250	1,598	38	12	0.700
**Age 14–18 yrs**	1,343	1,567	26	1,330	1,636	39	738	1,683	36	814	1,689	30	43	0.036**
Male	697	1,594	33	656	1,638	50	385	1,721	38	433	1,678	37	35	0.143
Female	646	1,535	31	674	1,625	36	353	1,644	36	381	1,698	37	54	0.036**
White, non-Hispanic	360	1,586	33	371	1,639	48	247	1,717	47	270	1,675	38	34	0.137
Black, non-Hispanic	488	1,542	42	450	1,531	27	195	1,594	50	174	1,609	25	27	0.137
Mexican-American	411	1,551	31	409	1,607	28	165	1,656	70	220	1,631	58	36	0.104
**Age 19–50 yrs**	2,583	1,657	17	2,845	1,717	20	2,865	1,718	14	3221	1,708	11	12	0.345
Male	1,226	1,651	21	1,302	1,687	22	1,404	1,712	15	1532	1,703	20	18	0.163
Female	1,357	1,660	25	1,543	1,742	29	1,461	1,723	22	1689	1,712	17	10	0.527
White, non-Hispanic	1,189	1,663	21	1,195	1,729	25	1,188	1,720	18	1410	1,709	15	11	0.432
Black, non-Hispanic	633	1,603	50	677	1,641	30	623	1,664	31	567	1,636	22	5	0.697
Mexican-American	560	1,578	17	716	1,598	25	598	1,602	15	672	1,601	30	9	0.113
**Age ≥51 yrs**	2,135	1,778	17	1,927	1,759	16	2,339	1,768	23	2,329	1,748	20	−8	0.159
Male	1,042	1,784	25	986	1,712	24	1,167	1,768	25	1,174	1,760	36	−3	0.904
Female	1,093	1,775	20	941	1,799	19	1,172	1,767	27	1,155	1,736	24	−15	0.290
White, non-Hispanic	1,280	1,799	18	1,136	1,771	17	1,134	1,752	17	1,139	1,738	25	−21	0.012**
Black, non-Hispanic	334	1,671	29	428	1,689	30	504	1,726	32	471	1,697	34	12	0.354
Mexican-American	399	1,637	45	275	1,567	47	347	1,657	42	410	1,631	31	4	0.809

* Sodium intake density was calculated as sodium intake divided by daily calories. Mean usual sodium intake density was estimated using PC-SIDE software (Department of Statistics, Iowa State University) with jackknife replicate weights and adjusted for the day of the week of the recall, age (years), sex, and race/ethnicity. Persons missing first-day recall data were excluded.

† Other racial/ethnic groups were not included. The sum of the sample size of non-Hispanic white, non-Hispanic black, and Mexican-American is not equal to the total sample size.

§ Sample sizes are unweighted.

¶ Mean change in sodium density per 2-year cycle (mg/1,000 kcal) estimated from a linear regression model with the usual mean sodium density for each 2-year phase weighted by the inverse of the variance.

** p<0.05, when mean usual sodium intake density was examined by using linear regression model.

## Footnotes

* Additional information available at http://www.cdc.gov/nchs/nhanes.htm.

† Additional information available at http://www.ars.usda.gov/ba/bhnrc/fsrg.

§ Additional information available at http://www.ars.usda.gov/services/docs.htm?docid=12089.

¶ Additional information available at http://www.ncbi.nlm.nih.gov/pubmed/23567248.

** The volume of human milk was assumed to be 600 mL per day for children aged 7–11 months fed only human milk; 600 mL per day minus the volume of infant formula plus other milk for other children aged 7–11 months, 89 mL per human milk feeding for children aged 12–18 months, and 59 mL per feeding for children aged 19–36 months. Sodium, potassium, and energy concentrations in human milk were assumed to be 177 mg/L, 531 mg/L, and 75 kcal/L, respectively, based on the USDA National Nutrient Database for Standard Reference values for mature, human milk, 33.8 fluid ounces per liter.

†† PC-SIDE (Software for Intake Distribution Estimation for the Windows operating system), Center for Agriculture and Rural Development, Iowa State University. Additional information available at http://www.side.stat.iastate.edu/pc-side.php and http://www.card.iastate.edu/publications/synopsis.aspx?id=168.

§§ Additional information available at http://www.cdc.gov/nchs/data/databriefs/db113.htm.

¶¶ Additional information available at http://www.gpo.gov/fdsys/pkg/FR-2012-01-26/html/2012-1010.htm.

*** Additional information available at http://www.scielosp.org/scielo.php?script=sci_arttext&pid=S1020-49892012001000009&lng=en&nrm=iso&tlng=en.
